# Study on triaxial compression performance and damage characteristics of fiber-reinforced ecological matrix cementing gangue gypsum fill material

**DOI:** 10.1371/journal.pone.0299001

**Published:** 2024-05-28

**Authors:** Qi Jiang, Zhigang Yin, Hang Yin, Runbo Ma

**Affiliations:** 1 School of Mining, Liaoning Technical University, Fuxin, China; 2 Chongqing University Liyang Smart City Research Institute, Liyang, China; 3 School of Civil Engineering, Liaoning Technical University, Fuxin, China; University of Duhok, IRAQ

## Abstract

Polypropylene fiber was equally mixed into alkali-activated slag fly ash geopolymer in order to ensure the filling effect of mine goaf and improve the stability of cemented gangue paste filling material with ecological matrix. Triaxial compression tests were then conducted under various conditions. The mechanical properties and damage characteristics of composite paste filling materials are studied, and the damage evolution model of paste filling materials under triaxial compression is established, based on the deviatoric stress-strain curve generated by the progressive failure behavior of samples. Internal physical and chemical mechanisms of the evolution of structure and characteristics are elucidated and comprehended via the use of SEM-EDS and XRD micro-techniques. The results show that the fiber can effectively improve the ultimate strength and the corresponding effective stress strength index of the sample within the scope of the experimental study. The best strengthening effect is achieved when the amount of NaOH is 3% of the mass of the solid material, the amount of fiber is 5‰ of the mass of the solid material, and the length of the fiber is about 12 mm. The action mode of the fiber in the sample is mainly divided into single-grip anchoring and three-dimensional mesh traction. As the crack initiates and develops, connection occurs in the matrix, where the fiber has an obvious interference and retardation effect on the crack propagation, thereby transforming the brittle failure into a ductile failure and consequently improving the fracture properties of the ecological cementitious coal gangue matrix. The theoretical damage evolution model of a segmented filling body is constructed by taking the initial compaction stage end point as the critical point, and the curve of the damage evolution model of the specimen under different conditions is obtained. The theoretical model is verified by the results from the triaxial compression test. We concluded that the experimental curve is in good agreement with the theoretical curve. Therefore, the established theoretical model has a certain reference value for the analysis and evaluation of the mechanical properties of paste filling materials. The research results can improve the utilization rate of solid waste resources.

## 1. Introduction

As the mining work progresses further into the goaf, the stability of the surrounding rock deteriorates due to the influence of the overlaying load, and the overlying rock commonly collapses [1,2]. To ensure the safety of underground mining, the filling mining method is an important way. However, the current global climate change problem is becoming more and more serious. The development of green cementitious materials to replace Portland cement with its serious energy consumption and large carbon dioxide emissions has become a research hotspot in the field of building materials. Alkali-activated materials have received extensive attention from researchers due to their low emissions and low energy consumption. Alkali-activated fly ash-slag polymer is a new type of hydraulic cementitious material formed by alkali-activated fly ash and slag [3–5]. Based on the excellent properties of geopolymer, such as short setting time, high compressive strength, acid and alkali corrosion resistance, and heavy metal ions, scholars at home and abroad (Puligilla, Li, Komnitsas, Temuujin) have made a lot of research reports on it [6–9]. It was found that when the geopolymer is stressed, it shows defects such as low tensile strength, high brittleness, and insufficient ductility [10]. These drawbacks seriously hinder the practical engineering application of alkali-activated materials. To improve the mechanical properties of geopolymers, many scholars have studied the strength characteristics of backfills with different matrix materials and solved practical engineering problems under the premise of satisfying the filling strength [11–13].

Zhou et al. [14] studied the mechanical properties of tantalum-niobium tailings cemented backfill under uniaxial compression and obtained the damage evolution law of the backfill. Xu et al. [15] investigated the internal relationship between energy dissipation and confining pressure, strain, and stress at various confining pressure loading stages and described the energy dissipation properties of cemented backfill under triaxial compression. The effect of mechanical activation on the cementitious activity of ultrafine tailings, as well as the filling performance and hydration mechanism of non-clinker filler bodies, was discussed by Zhang et al. [16]. Sun et al. [17,18] excited fly ash as cementing material to prepare fly ash geopolymer gangue aggregate filling body, and analyzed the deformation localization law of this filling body by digital speckle correlation method. This formula can reduce the dosage of cement. He et al. [19] used electrolytic manganese slag and barium slag to prepare tourmaline-sulphoaluminate cement. Wang et al. [20] verified a new method of mine deep cooling based on PCM backfill by numerical simulation and experiment. Deng et al. [21] revealed the mechanism of the strength of cement backfill influence on the filling rate through laboratory tests and theoretical analysis, expounded the calculation principle of the target filling rate of multi-scene cement backfill, and finally formed the dynamic design method of the strength demand of cement backfill guided by the filling rate control in coal mine goaf. Zhao Kang et al. [22], Liu L et al. [23], Zhao Kui et al. [24] carried out theoretical research on the strength of backfill under different conditions, and achieved a series of results. Xu Wenbin et al. [25], Li E et al. [26], Xu Jun et al. [27] through a large number of experimental studies show that: fiber can enhance the strength of the filling body, the influencing factors are fiber type, content and fiber length. Sun et al. [28] found that PVA fiber can improve the brittleness of fly ash-based geopolymers and enhance their toughness. Cao et al. [29] studied the strength, toughness, and microstructure characteristics of the filling body with three different types of fiber. The results showed that the toughness of the filling body changed significantly with the addition of different fiber types and contents, and the fiber content affected the strength performance of the filling body. Dias et al. [30] investigated the fracture toughness of basalt fiber-reinforced geopolymer concrete and compared it to standard Portland cement concrete reinforced with basalt fiber. The fracture toughness of geopolymer concrete was found to be greater than that of standard Portland concrete. Kinga Korniejenko et al. [31] focused on the use of polymer short fibers and the mechanical properties of geopolymer composites. R. Subalakshmi et al. [32] research shows that foam concrete utilized in GFRG panels has desirable strength, making it a suitable alternative construction material for the industrialized building system. Kamran tavakol et al. [33] conducted a series of direct shear tests to evaluate the effects of cement, rice husk ash, polypropylene fiber and relative density on the mechanical properties of calcareous sand. Seyed Hadi Sahlabadi et al. [34] research shows that the strength of the PPF-reinforced specimens is significantly more than that of BF-reinforced ones.

The above research results have promoted the development of mine filling materials. However, there are few studies on the strength forming mechanism of polymer and the damage evolution characteristics of paste filling materials under triaxial compression. In this paper, aiming at the engineering problems of poor surrounding rock stability and large safety hazards in the goaf of metal mines, fly ash, slag, and coal gangue are used as raw materials to cementedly fill the underground goaf as the background, and the purpose of improving the defects of high brittleness, poor toughness, and easy cracking of geopolymers is to incorporate polypropylene fiber into it. The mechanical properties of ecological matrix-cemented gangue gypsum filling materials were studied by indoor triaxial compression testing, SEM-EDS, and XRD microscopic techniques. Based on the deviatoric stress-strain curve generated by the progressive failure behavior of the paste filling material, a segmented damage evolution model with the initial compaction stage of the sample as the critical point is established. These results of this study can help solve the problem of environmental pollution caused by the accumulation of industrial solid waste materials as well as provide a theoretical basis for goaf filling and promoting sustainable development.

## 2. Test materials and methods

### 2.1 Testing materials

**Fig 1:**
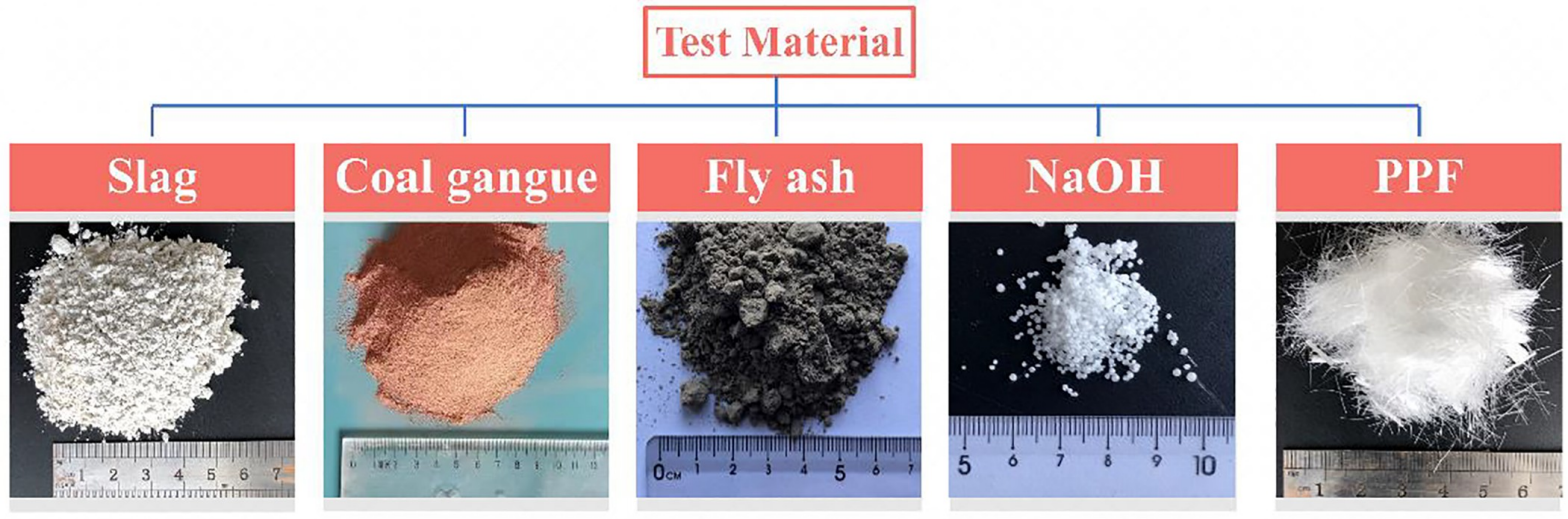
Test materials.

The test materials include blast furnace slag, fly ash, coal gangue, NaOH particles, water, and polypropylene fiber. The blast furnace slag is S95-grade slag produced by Longze Water Purification Material Co. Ltd., Gongyi City, Henan Province. The chemical composition of the slag is obtained via X-ray fluorescence (XRF) spectrometry, as shown in Table 1. The fly ash––F class II fly ash below C50––was provided by the Tangshan Branch of Datang Tongzhou Technology Co. Ltd. The main chemical composition and performance indicators are shown in Table 2. The coal gangue was acquired from the gangue dump of China Shenhua Energy Co. Ltd., Shendong Coal Branch. The chemical composition is shown in Table 3. After on-site sampling is completed, the coal gangue is returned to the laboratory for crushing. The crushed coal gangue was placed in a mechanical grinder to obtain a fine-grained solid waste spontaneous combustion coal gangue powder with a particle size of less than 1.18 mm. Sodium hydroxide solid particles were provided by Liaoning Quanrui Reagent Co. Ltd. with a purity of approximately 96%. Tap water from Fuxin City was used for the test; polypropylene fiber was provided by Senxiang Building Materials Company in Langli Street, Changsha County; the physical and mechanical properties are shown in Table 4. The sample raw material properties are shown in Fig 1.

**Table 1:** Chemical composition of slag.

material	CaO	Fe_2_O_3_	Al_2_O_3_	SiO_2_	MgO	SO_3_	K_2_O	MnO	TiO_2_	Na_2_O	Others
slag	49.411	0.27	13.1	26.223	5.16	2.341	0.336	0.426	2.133	0.413	0.187

**Table 2:** Main chemical composition and performance index of fly ash.

Indicators	Fineness (80) / %	Water requirement ratio / %	Burning vector / %	Chloride ion / %	Water content / %	SO/%	Calcium sulfite hemihydrate	CaO/%	Free CaO/%	Total mass fraction of SiO2, Al2O3 and Fe2O3	Density /(g·cm^-3^)
Numerical value	15~30	102~105	2.10	0.01	0.70	1.84	2%	5.90	0.40	73	1.8~2.2

**Table 3:** Chemical composition of coal gangue (%).

material	SiO_2_	Fe_2_O_3_	Al_2_O_3_	CaO	MgO	SO_3_	TiO_2_	K_2_O	Na_2_O
coal gangue	45.75	5.10	13.01	3.69	1.06	0.76	0.66	1.80	0.94

**Table 4:** Physical Properties of Polypropylene Fiber.

Type	Density/(g·cm^-3^)	Tensile strength/MPa	Elastic modulus/GPa	Melting point/°C	Ignition point/°C	Fracture elongation /%	Dispersibility
Bundle monofilament	0.91	≥300	≥3.5	165	590	≥15	Fabulous

### 2.2 Test method

In this experiment, blast furnace slag and fly ash were used for reaction in an alkaline environment for forming a hydraulic cementitious matrix. Fine-grained coal gangue was used as aggregate, and polypropylene fiber was used as reinforcing material to prepare fiber-reinforced ecological matrix-cemented gangue-gypsum fill material. During the test, on the basis of the research in reference [35], three influencing factors—NaOH content, fiber content, and fiber length—were considered. Six levels were designed for each influencing factor, and a total of 19 groups of tests were designed. Group D was the reference group. The test design scheme is shown in Table 5. Weight of solid material: W, Kg; weight of slag: W_1_, Kg; weight of fly ash: W_2_, Kg; weight of coal gangue: W_3_, Kg; weight of water: W_4_, Kg; weight of NaOH: W_5_, Kg; fiber weight: W_6_, Kg; mass concentration: MC. Where W = W_1_ + W_2_ + W_3_; the slag content is 0.1 W; the fly ash content is 0.5 W; coal gangue content is 0.4 W; M_c_ = W / (W + W_4_); the content of NaOH is equal to the ratio of W_5_ to W; and the fiber content is equal to the ratio of W_6_ to W.

**Table 5:** Test plan.

Group	Sample number	Slag content/%	Fly ash content/%	Coal gangue content/%	Mass concentration	NaOH content/%	Fiber content/‰	Fiber length/mm
A	A1	10	50	40	70	0.5	5	12
	A2					1.0		
	A3					1.5		
	A4					2.0		
	A5					2.5		
	A6					3.0		
B	B1	10	50	40	70	2.0	3	12
	B2						4	
	B3						5	
	B4						6	
	B5						7	
	B6						8	
C	C1	10	50	40	70	2.0	5	3
	C2							6
	C3							9
	C4							12
	C5							15
	C6							20
D	D0	10	50	40	70	2.0	0	0

**Fig 2:**
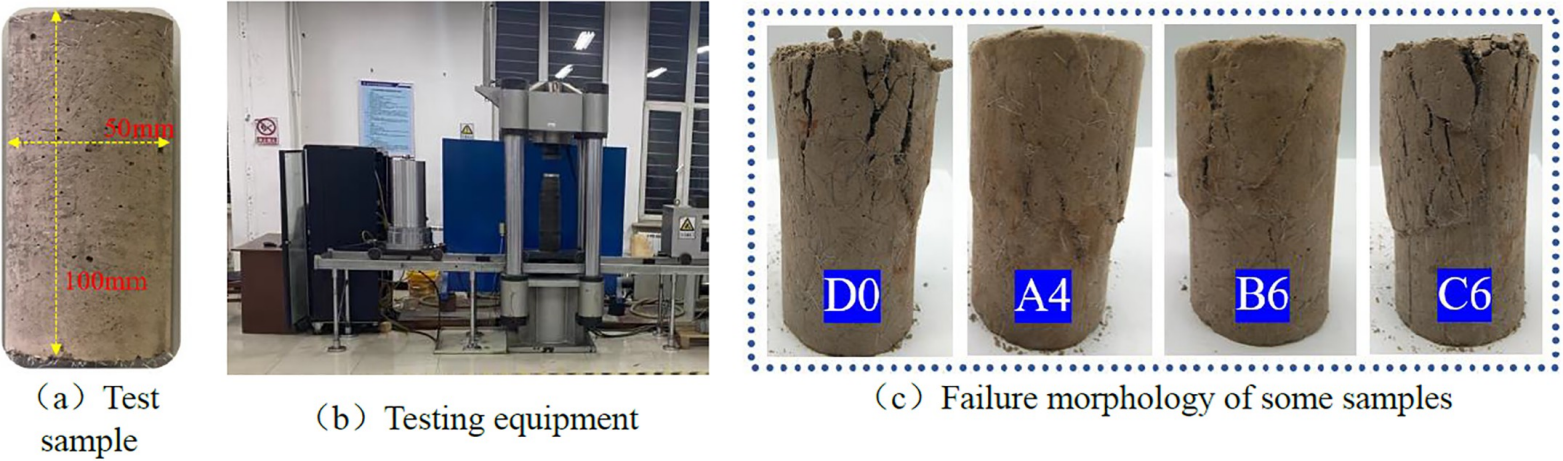
Loading instruments and samples.

According to the aforedescribed test plan, the raw materials needed in the sample preparation process were weighed in turn. The water was first mixed uniformly after the sodium hydroxide granules had been added. Considering the impact of temperature on the test and the fact that the sodium hydroxide particles were exothermic throughout the dissolution process, an alkali-activated solution was created and chilled to room temperature before use. (The cooling time is 24 hours) Second, the weighed slag, fly ash, and coal gangue were mixed and stirred evenly, then slowly poured into the mortar mixer for stirring for 3–5 minutes. Thereafter, the cooled alkali activator was added for stirring. The evenly mixed paste was placed in a 50 mm ×100 mm mould and vibrated for 30 s. After curing at room temperature for 24 h, the mold was removed. Finally, the samples were placed in a standard curing box for curing at a temperature of 20°C ±2°C, the humidity was kept at approximately 90%, and the curing time was 28 days. The specimen reaching the last day of curing age is shown in Fig 2(a).

TAW-2000 electro-hydraulic servo rock triaxial testing machine was used to carry out the corresponding triaxial compression test. The whole test process was automatically controlled by a computer, and the relevant test data were recorded. The test adopted the shear rate was 0.4 mm/min, and the confining pressures were set to 50, 100, and 200 kPa. The test was repeated until the deformation reaches 10% after reaching peak strength and stopped, The failure modes of some samples are shown in Fig 2(c).

**Fig 3:**
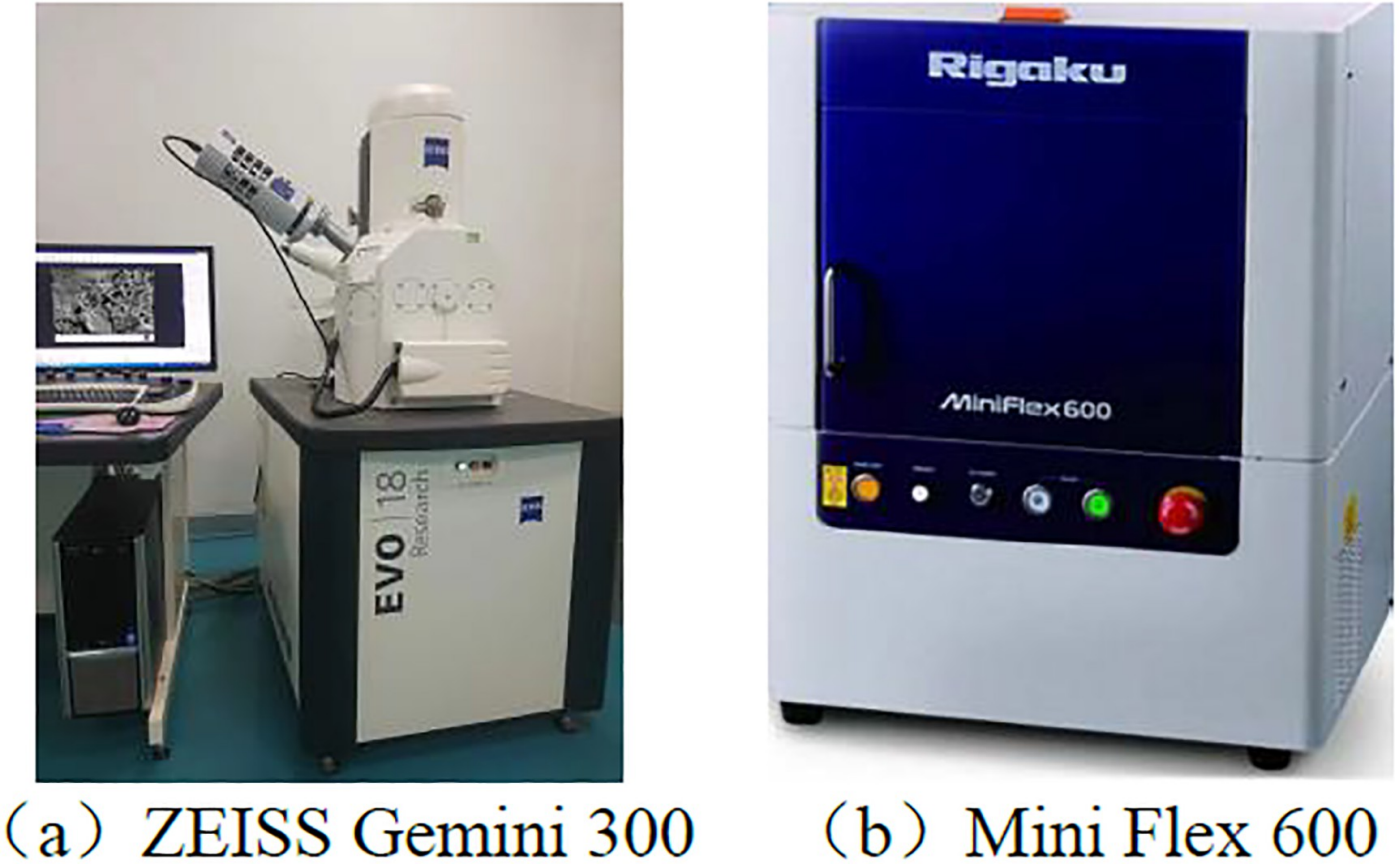
Microscopic test equipment.

After the triaxial compression test is completed, small pieces with the size of about 5mm×5mm×5mm are cut from the shear zone of the sample, dried in a drying box for 24 hours, and then treated by gold spraying. ZEISS Gemini 300 field emission scanning electron microscope was used to analyze the contact interface between bulk particles and hydration products, fiber failure mode and micro-morphology of eco-gel matrix. The acceleration voltage of the equipment was 20V~30Kv, and the secondary electron resolution was 1.2 nm at 1 kV and 0.7 nm at 15 kV. The samples were analyzed using an Oxford Xplore 30 energy spectrometer. X-ray diffraction (XRD) was used to test the mineral composition of the eco-gel matrix with a Mini Flex 600 X-ray diffractometer. The sample tested by XRD has a length and width of 1-2cm (generally not less than 1cm) and a thickness of not more than 15mm, and it is required to be ground in a mortar with a particle size of 320 meshes, about 40 microns. The test equipment is shown in Fig 3.

## 3. Test results and analysis

### 3.1 Triaxial compression test results

**Fig 4:**
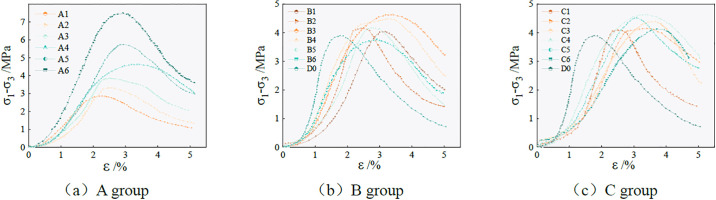
The deviatoric stress-strain curve of the specimen.

According to the established test plan, triaxial compression tests were carried out on samples with different ratios. A cure age of 28 days was recorded at confining pressures of 50, 100, and 200 kPa, and the relationship between deviatoric stress and strain was recorded. One hundred and seventy-one deviatoric stress-strain data sets were obtained under different conditions. In the range of the experimental study, the deviatoric stress value of the sample increases with the increase in confining pressure. The deviatoric stress-strain curve obtained when the confining pressure was 200 kPa is shown in Fig 4. The typical failure modes of the specimens were observed, as shown in Fig 2(c). When the confining pressure is 200 kPa, the D0 sample undergoes compression failure, the top block of the sample peels off to a large extent, and vertical cracks appear on the edge. When the axial load of the sample reaches the critical value, yield and failure begin to occur inside the sample. The sample gradually deforms, produces strong plastic deformation, and finally presents an unstable failure state. After adding 5–12 mm of fiber into the sample, the failure mode is like A4 in Fig 2(c). The sample can basically maintain the original shape without spalling. When the shear stress reaches a critical value, the yield and failure begin to occur inside the sample, and the shear crack occurs in the sample, but the through-crack surface is not formed. After adding excessive fiber, the sample B6 also has vertical cracks on the edge after loading, and the overall failure is a wedge-shaped failure. After the incorporation of longer fibers, after the loading of the sample C6, due to the intertwining of the fibers in the sample, a weak structure is formed, and the bonding force of the matrix is reduced. Therefore, the damage to the sample is more serious, and there is obvious matrix shedding and crack penetration.

From the curve in Fig 4, the deviatoric stress-strain curves of the samples under different conditions exhibit a similar yield mode. In selecting the yield point, the stress at the first peak and decrease are considered the ultimate stress corresponding to the curve, while the strain value corresponding to the ultimate stress is the ultimate strain curve. In the group A test, when the axial strain is small, the deviatoric stress-strain curve of the sample basically coincides. With the increase in axial strain, the greater the NaOH content, the greater the deviatoric stress value of the sample, but the deviatoric stress-strain curve of the sample with a smaller NaOH content after reaching the peak load is relatively gentle. In the group B test, the deviatoric stress-strain curve is strain softening, and the deviatoric stress increases first and then decreases with the increase of strain, and finally tends to be stable. However, the ultimate strain corresponding to the peak load of the sample is also different when the fiber content is different. When the sample is mixed with 5–12 mm of fiber, the deformation that the sample can withstand is significantly increased compared with that of the sample without fiber, indicating that the addition of an appropriate amount of fiber to the sample can effectively improve its toughness. In the group C test, as the fiber length increases, the ultimate strain value of the sample gradually increases, indicating that the appropriate amount of fiber is added to the sample, and the failure of the sample gradually changes from brittle failure to ductile failure.

Elastic modulus is an important engineering material performance parameter. From a macro perspective, it is a measure of the ability of an object to resist elastomeric deformation. It is a micro-reflection of the bonding strength between atoms, ions, or molecules. As an index reflecting the material’s ability to resist elastic deformation, during the test, the slope of the almost straight-line segment of the deviatoric stress-strain curve before the ultimate stress is used to represent the slope. The first-order linear derivation of the secant lines of the peak stress at 30 and 70% in the deviatoric stress-strain curve is carried out using the Origin software. Hence, the elastic modulus of the sample is obtained, and the elastic modulus E is calculated according to formula (1) as expressed below:

$$E=\frac{\Delta \sigma }{\Delta \varepsilon }$$ (1)

In the formula: Δ*σ* is the change of 30% and 70% of the ultimate stress; Δ*ε* is the difference between the axial strain of 30% and 70% of the ultimate stress.

To better evaluate the reinforcement effect of fiber on the ecological matrix cemented gangue gypsum filling material, the strength changes before and after the fiber incorporation are compared, and the reinforcement effect coefficient R is introduced when the sample’s strength is at its maximum.


$$R=\frac{{\left(\sigma_{1}-\sigma_{3}\right)}_{f}}{\left(\sigma_{1}-\sigma_{3}\right)}$$ (2)

In the formula: (*σ*_1_ − *σ*_3_)_*f*_ is the ultimate strength of the specimen after adding fiber; (*σ*_1_ − *σ*_3_) is the ultimate strength of the specimen without fiber.

### 3.2 Effect analysis of influencing factors

**Fig 5:**
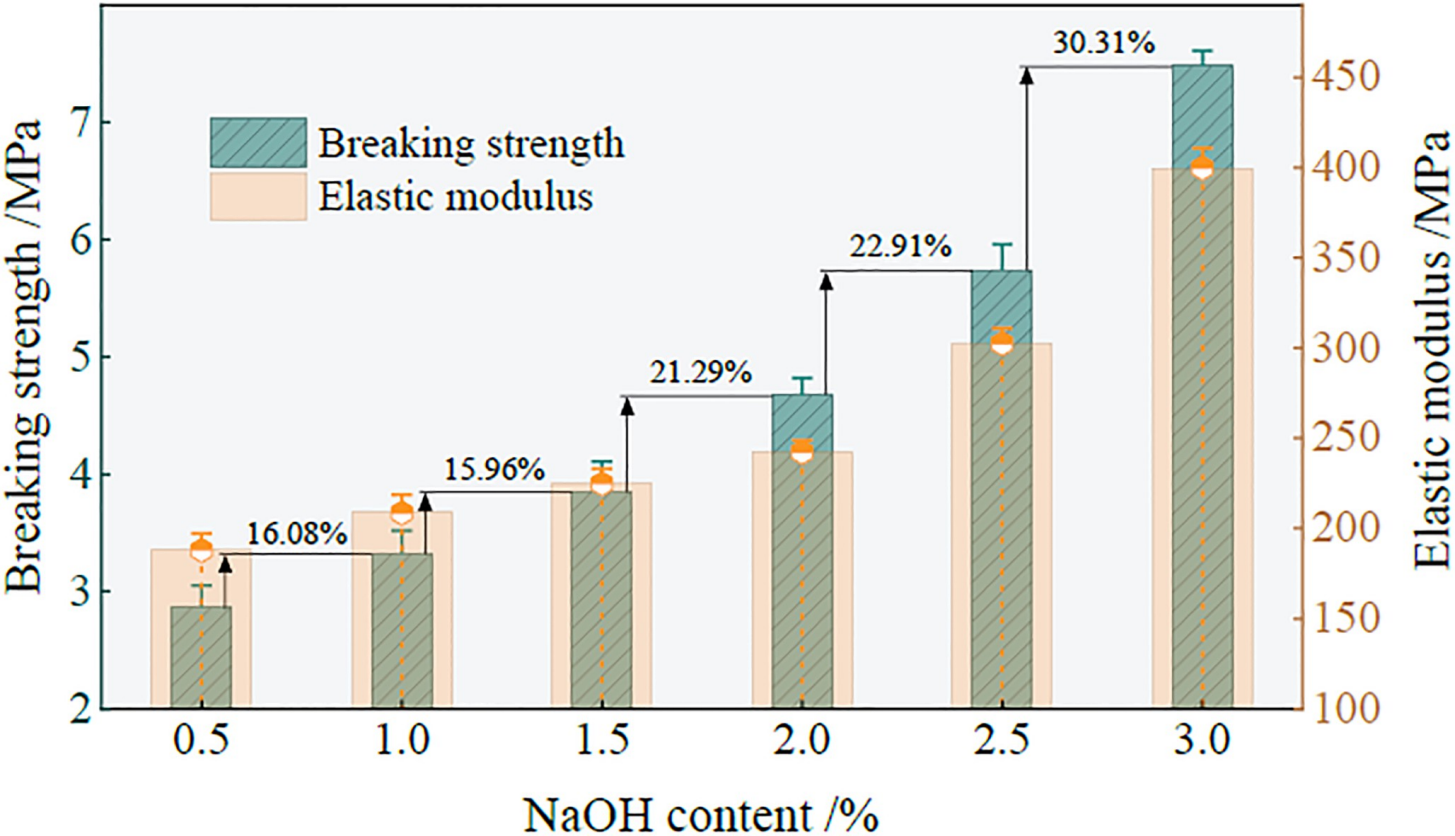
The relationship between the ultimate strength, elastic modulus and NaOH content of the sample.

Based on the above calculation, the relationship between the ultimate strength, elastic modulus, and NaOH content in group A is shown in Fig 5.

With the increase in NaOH content, the ultimate failure strength and elastic modulus of the sample show a slow growth trend. When NaOH is 3%, the ultimate failure strength of the sample reaches the local maximum. Compared to a NaOH content of 0.5%, the ultimate failure strength increases by 4.62 MPa. This is because when the NaOH content is small, the driving force of the polymerization reaction process is insufficient, and the degree of polymerization of the generated product is small, resulting in the structure of the material after hardening. With the increase in NaOH, the pH in the polymerization reaction environment increases. The higher PH solution environment promotes the dissolution of Ca, Si, Al, and other elements so that more gray bodies dissolve to form silicate and aluminate monomers, accelerating the polymerization reaction speed and making the reaction more intense, thus generating more hydraulic cementitious matrix, and increasing the ultimate failure strength.

**Fig 6:**
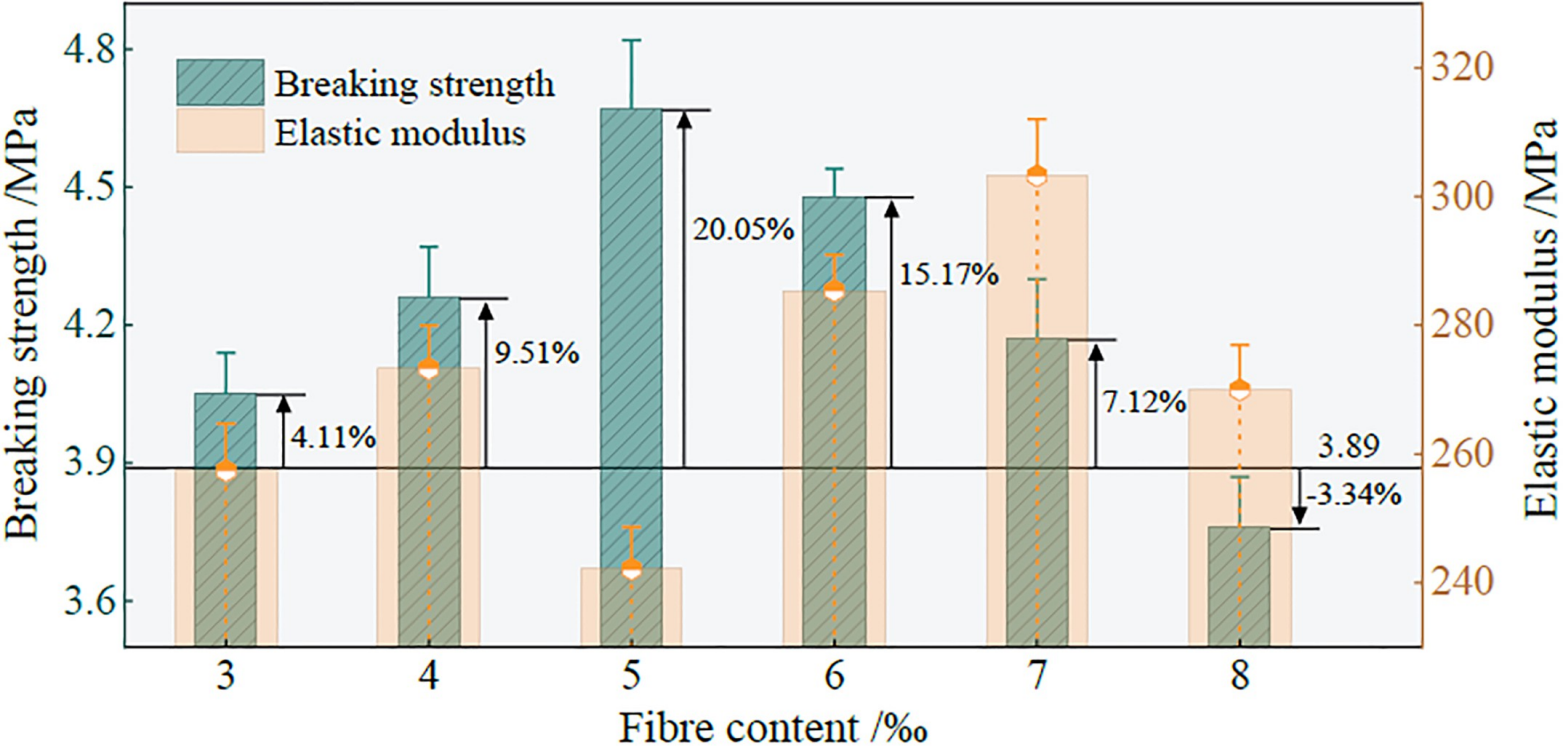
The relationship between the ultimate strength, elastic modulus, reinforcement effect coefficient and fiber content of the sample.

Based on the above calculation, the relationship between the ultimate strength, elastic modulus, reinforcement effect coefficient, and fiber content in group B is shown in Fig 6.

Fig 6 shows that with an increase in fiber content, the ultimate strength of the sample at the time of failure initially increases and then decreases. The ultimate strength of the sample reaches the local maximum when the fiber content is 5, which is 20.05% higher than that of the sample without fiber. When the fiber content is 8, the ultimate strength of the sample is 3.34% lower than that with no fiber content. There-fore, the addition of an appropriate amount of fiber can effectively improve the ductil-ity of the ecological matrix cemented gangue gypsum fill material. The elastic modulus of the sample varies with increasing fiber content in the range of experimental re-search. However, when the fiber content is 5, the elastic modulus of the sample is the lowest, indicating that the elastic deformation is large and the ductility of the material is good before the sample reaches the ultimate strength failure. When the sample is subjected to triaxial compression, the micro-voids inside the sample are gradually connected, and micro-cracks are developed. When the micro-cracks are distributed between the fibers, due to the random occurrence of an appropriate amount of fibers inside the sample, they intersect with the hydraulic cementitious matrix to form a three-dimensional network structure. Subsequently, the internal stress field of the sample tends to be uniform through the bridging effect. The stress concentrated at one end of the crack is alleviated, inhibiting further development of the micro-cracks, which makes crack propagation difficult. When the fiber content exceeds the critical value, the fiber agglomerates in the sample, and a weak surface of the hole is formed at the bonding surface of the hydraulic cementitious matrix. This reduces the bridge stress between the fiber and the hydraulic cementitious matrix, results in the formation of internal defects in the sample matrix, and lowers the damage resistance of the material. However, in reference [35], researchers have come to the conclusion that there is an optimal fiber content of 6‰ in the fiber-reinforced solid waste polymer filling material, because the fiber length used in this study is 9mm when the compressive strength is the highest.

**Fig 7:**
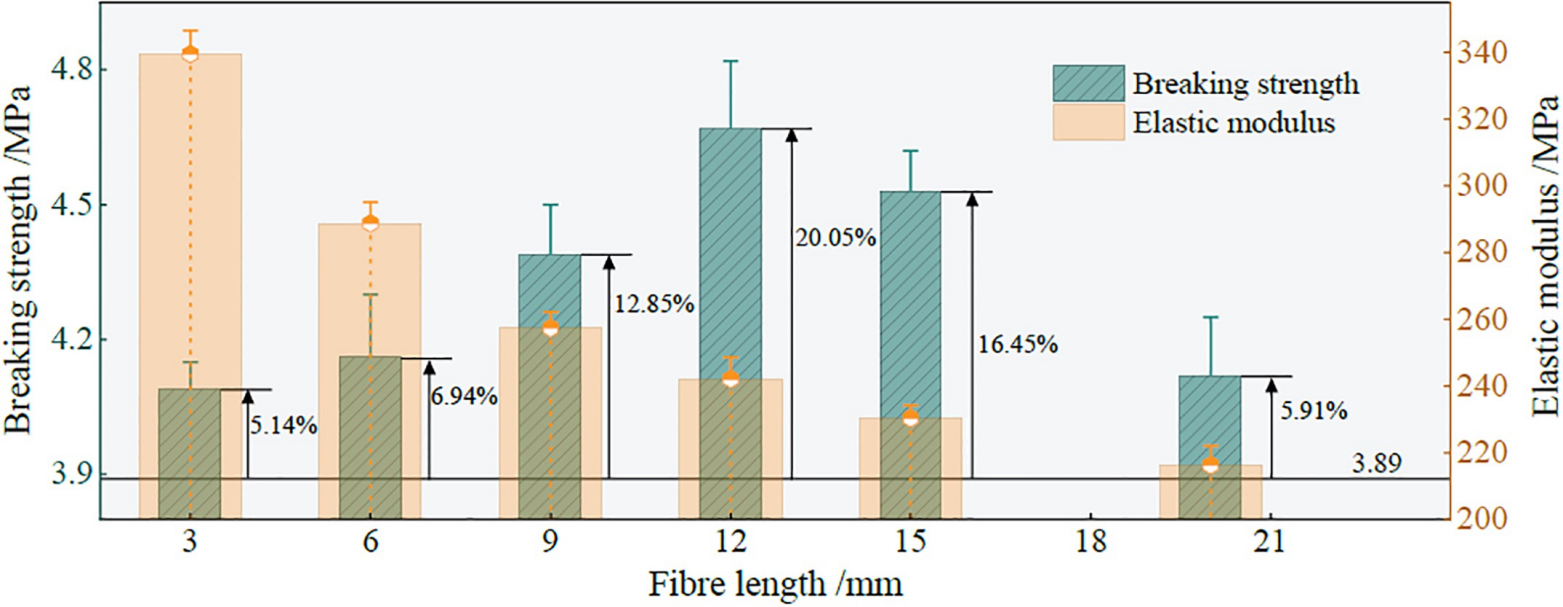
The relationship between ultimate strength, elastic modulus, reinforcement effect coefficient and fiber length.

Based on the above results, the relationship between the ultimate strength, elastic modulus, reinforcement effect coefficient, and fiber length in group C is shown in Fig 7.

Fig 7 shows that within the scope of the study, the ultimate failure strength of the sample initially increases and then decreases with an increase in the fiber length, and the elastic modulus gradually decreases. When the fiber length is short, the inter-face between the fiber surface and the hydraulic cementitious matrix can be weakened and separated easily during crack propagation, leading to the failure of the specimen. With increasing fiber length, the fiber surface and the hydraulic cementitious matrix form an effective bond. When the specimen is subjected to triaxial compression, the fiber and the hydraulic cementitious matrix share the strain together, effectively delaying the unstable propagation phase of the crack. When the same quality fiber is mixed into the sample, the number of fibers decreases with an increase in fiber length. When the sample is loaded, the granular particles tend to slide relative to the fiber and pro-duce friction. The fiber remains in its elastic working state, and increase in its deformation evolves with the change of the macroscopic stress acting on the surface of the sample. When the interaction force between the fiber and the cementitious matrix interface is greater than the tensile strength of the fiber, the crack resistance effect of the polypropylene fiber on the sample fails, and the fracture occurs [36].

### 3.3 Analysis of shear strength parameters

According to the results of the triaxial compression test, the Mohr’s circle under the effective stress state is drawn when the specimen is destroyed, and the common tangent of the Mohr’s circle under different confining pressures (50 kPa, 100 kPa, and 200 kPa) is obtained. This common tangent corresponds to the effective stress intensity envelope of the specimen.

The fitted common tangent is represented by *τ* = *σ*tan*φ*′+*c*′, and the effective stress intensity index (effective cohesion *c*′ and effective internal friction angle *φ*′) of the sample under each ratio can be calculated. The specific results are shown in Table 6.

**Table 6:** Effective stress strength index of specimens.

Sample number	*c*′/kPa	*φ*′/°	Sample number	*c*′/kPa	*φ*′/°	Sample number	*c*′/kPa	*φ*′/°
A1	305.11	38.79	B1	482.31	37.47	C1	450.68	36.05
A2	359.62	37.54	B2	516.22	38.03	C2	483.34	37.04
A3	428.59	35.21	B3	530.74	36.85	C3	509.83	35.92
A4	530.74	36.85	B4	507.82	35.88	C4	530.74	36.85
A5	649.14	35.98	B5	475.11	36.32	C5	523.37	37.79
A6	790.77	37.19	B6	437.95	37.09	C6	492.79	37.24
						D0	446.34	36.39

**Fig 8:**
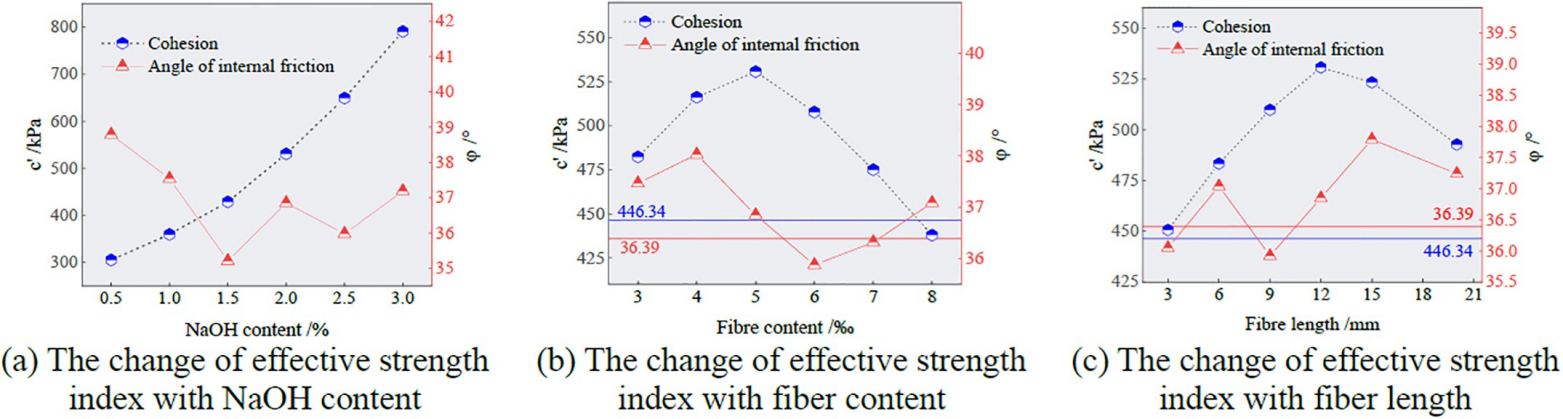
Change of effective strength index.

According to the data in Table 6, the relationship between the effective stress strength index of the sample and the NaOH content, fiber content and fiber length is drawn, as shown in Fig 8.

As shown in Fig 8(a), the effective cohesion of the sample increases as the NaOH content increases. The effective cohesion of the sample reaches the maximum when NaOH content is 3%. It can be seen from Fig 8(b) and 8(c) that with increasing fiber content and length, the effective cohesion of the sample first increases and then decreases. When the fiber content is 5 and the length is 12 mm, the effective cohesion reaches the maximum of 530.74 kPa. Appropriate fibers are evenly distributed inside the sample, and the contact area with the polymer matrix increases, which enhances friction and cementation. During the shearing process, many fibers bear tension, which inhibits the damage of the sample to a certain extent, thereby improving the cohesion of the sample. When the fiber content exceeds 5‰, the cohesion decreases to a certain extent. This phenomenon is due to the uneven distribution of too many fibers in the interior, which may have been partially agglomerated or penetrated into cracks, resulting in a decrease in the degree of occlusion between the fiber and the ecological matrix. Therefore, the increase in cohesion is not obvious or even more easily damaged. The incorporation of fibers has little effect on the roughness and staggered degree of the ecological matrix, so it has little effect on the internal friction angle. The shear strength of the ecological matrix is determined by the cohesion and internal friction angles. Therefore, the improvement of the strength of the ecological matrix cemented gangue by fiber reinforcement can be considered to be achieved by enhancing the overall cohesion.

From Fig 8(c), it can be seen that with the continuous increase in fiber length, the cohesion of fiber aeolian sand increases continuously. Compared with the D0 sample, the cohesion of the C4 sample increases by about 84.4 kPa. When the fiber length exceeds 12 mm, the cohesion of the sample begins to decrease. The reason for this phenomenon is that the fiber and the ecological matrix are intertwined to form a spatial skeleton structure. As the fiber length increases, the fiber and the ecological matrix produce greater interaction, which enhances the anchoring effect of the fiber and plays a good role in constraining the deformation of the sample. The variation amplitude of the internal friction angle does not change significantly with the fiber length and almost fluctuates around the internal friction angle of the D0 sample.

### 3.4 Microscopic characterization analysis

**Fig 9:**
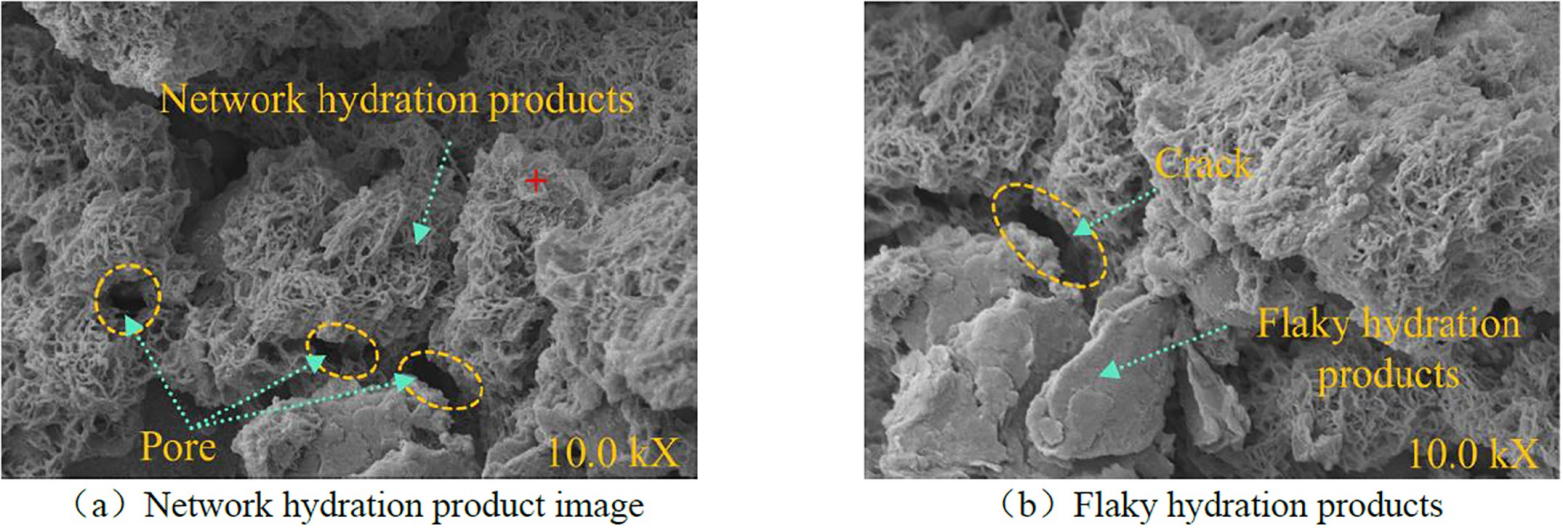
Microstructure of sample.

The microstructure of the sample(B4) after triaxial compression was observed by scanning electron microscopy, and different positions of the sample were observed, respectively. The scanning results are shown in Fig 9.

Performance of the material of the structure is the fundamental factor affecting the macroscopic mechanical properties and failure mechanism of the structure. The performance of the material mainly depends on the composition and failure mechanism of the fine microstructure inside the material. As shown in Fig 9(a) and 9(b), when the sample is cured over 28 d, the internal accumulation of the filling material forms a large number of honeycomb network polymerization products. As shown in Fig 9, the honeycomb network polymerization product is in the gap of continuous particle gradation. When subjected to external load, the honeycomb topology formed by the honeycomb network polymerization product can effectively disperse the external force in all directions, so that the mixed material has good structural stability and good mechanical properties. During the early stage of damage, as a deformation aggregate containing continuous distribution defects, the defects such as micro-cracks and micro-voids in the ecological cementitious coal gangue matrix are random. During the condensation and hardening of the sample, water evaporates from the pores, forming an unsaturated area of the polymerization product and resulting in pore defects in the material.

In the NaOH excitation solution, the covalent bonds such as high-energy silicon-oxygen bond (-Si-O-) and aluminum-oxygen bond (*-Al-O-*) in the silicon-aluminum material are broken, and the reaction process is shown in Eqs (3) and (4).


$$n(2SiO_{\text{2}}\cdot Al_{\text{2}}O_{\text{3}})+4H_{\text{2}}O\overset{NaOH}{\to }n{\left(OH\right)}_{\text{3}}-Si-O-Al-O-{\left(OH\right)}_{\text{3}}$$ (3)

$$n{\left(OH\right)}_{3}-Si-O-Al-O-{\left(OH\right)}_{3}\overset{NaOH}{\to }Na(-Si-O-Al-O-Si-)+4H_{2}O$$ (4)

During the reaction process, the product in formula (3) further acts as the reactant in formula (4), and finally produces a stable three-dimensional polyaluminate structure polymer product. The aluminate polymerization products formed by the reaction are continuously interwoven, polymerized, dehydrated, and hardened to form a three-dimensional structure with short-range order and long-range disorder. In the three-dimensional structure, *Al*^3+^ replaces *Si*^4+^ to occupy the position of silicon ions, forming a structure of [*SiO*_4_]^4-^ and [*AlO*_4_]^5-^ connected by oxygen atoms. Since *Al*^3+^ is an ion with a positive trivalent ion, it has a negative charge around it. In order to balance the electricity price, alkali ions such as potassium and sodium with positive charges are attracted to the channel of the gel. When exchanging with other ions, it will not cause structural damage due to the loss of alkali ions, thereby obtaining a relatively stable amorphous or quasi-crystalline three-dimensional network structure of inorganic polymers.

**Fig 10:**
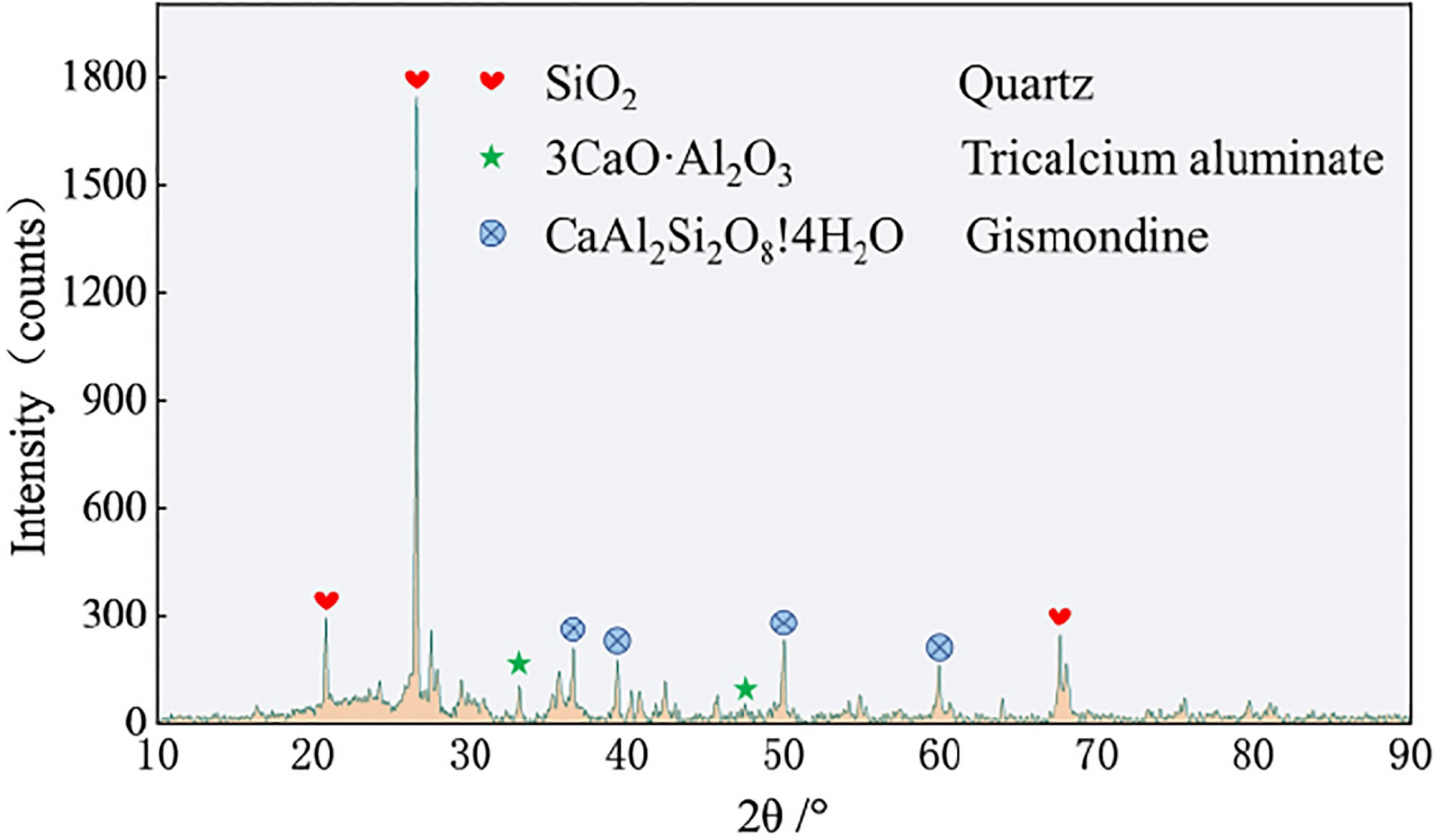
XRD diffraction patterns of samples.

The mineral composition of the sample was analyzed by XRD. The XRD pattern is shown in Fig 10. The figure shows that more diffraction peaks are present in the range of 20°–40°, indicating the presence of many amorphous silicate gels in the product [37]. Furthermore, the sample exhibits a diffraction peak at approximately 26.7° which corresponds to that of silica based on the comparison with the PDF card. The diffraction peak appearing at approximately 50° is relatively stable, which corresponds to calcium aluminosilicate hydrate based on the comparison with the PDF card.

**Fig 11:**
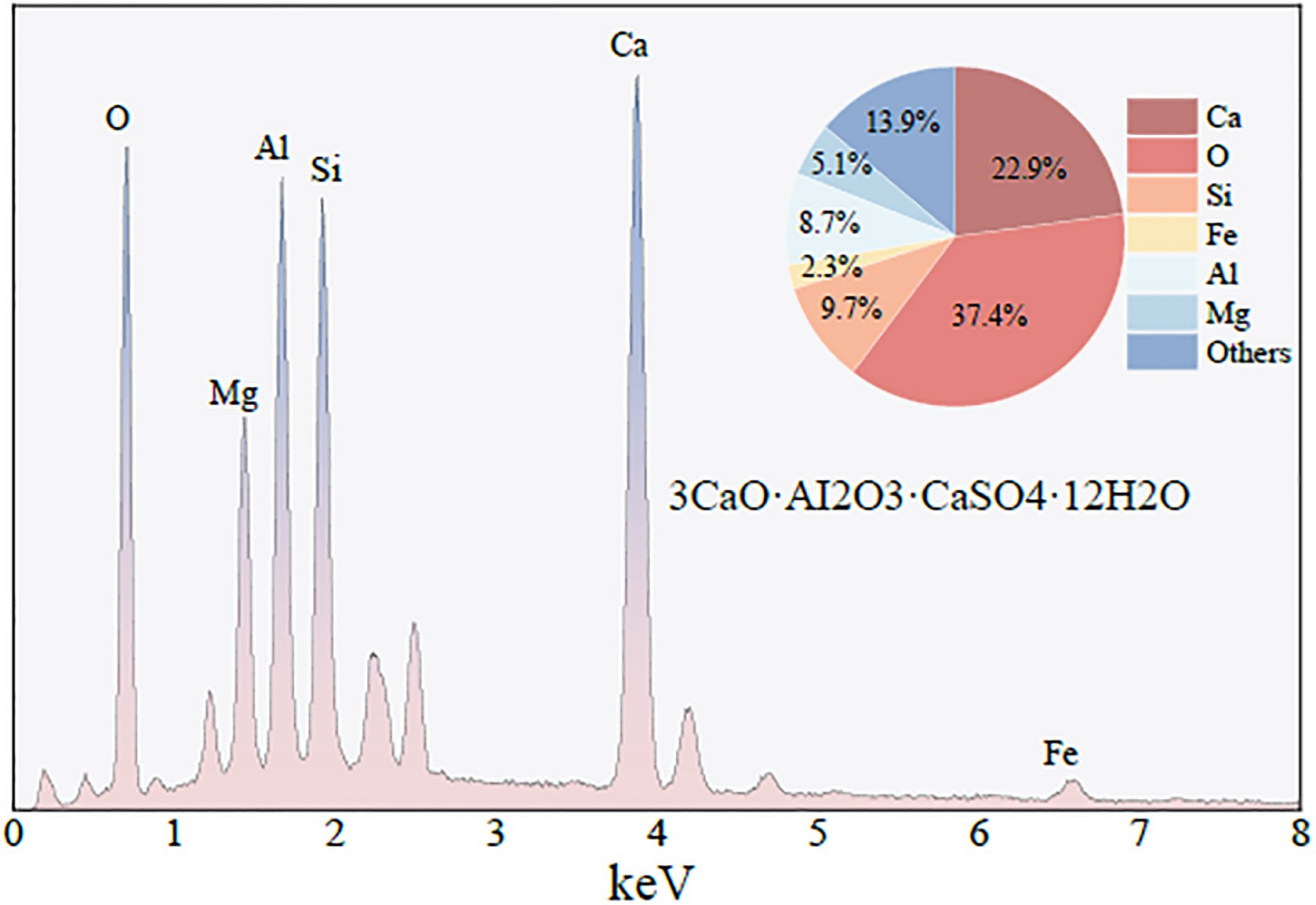
EDS energy spectrum of hydration products.

In the product (Fig 9(a)), a point is selected for EDS energy spectrum analysis. As shown in Fig 11, the main elements contained in the hydration product are: Ca, O, Si, Al, etc., Among them, the content of Ca element is 22.9%, the content of O element is 37.4%, the content of Si element is 9.7%, and the content of Al element is 8.7%. It can be judged from the constituent elements that the reaction product is *C* − *A* − *S* − *H* gel. The *C* − *A* − *S* − *H* gel is formed by the *Si* − *O* − *Si* and *Al* − *O* − *Al* covalent bonds in coal gangue, which are depleted and broken under the action of NaOH to form $SiO_{4}^{4-}$ and $AlO_{4}^{5-}$, and combined with the *Ca*^2+^ generated by the dissolution of slag under the action of alkali.

**Fig 12:**
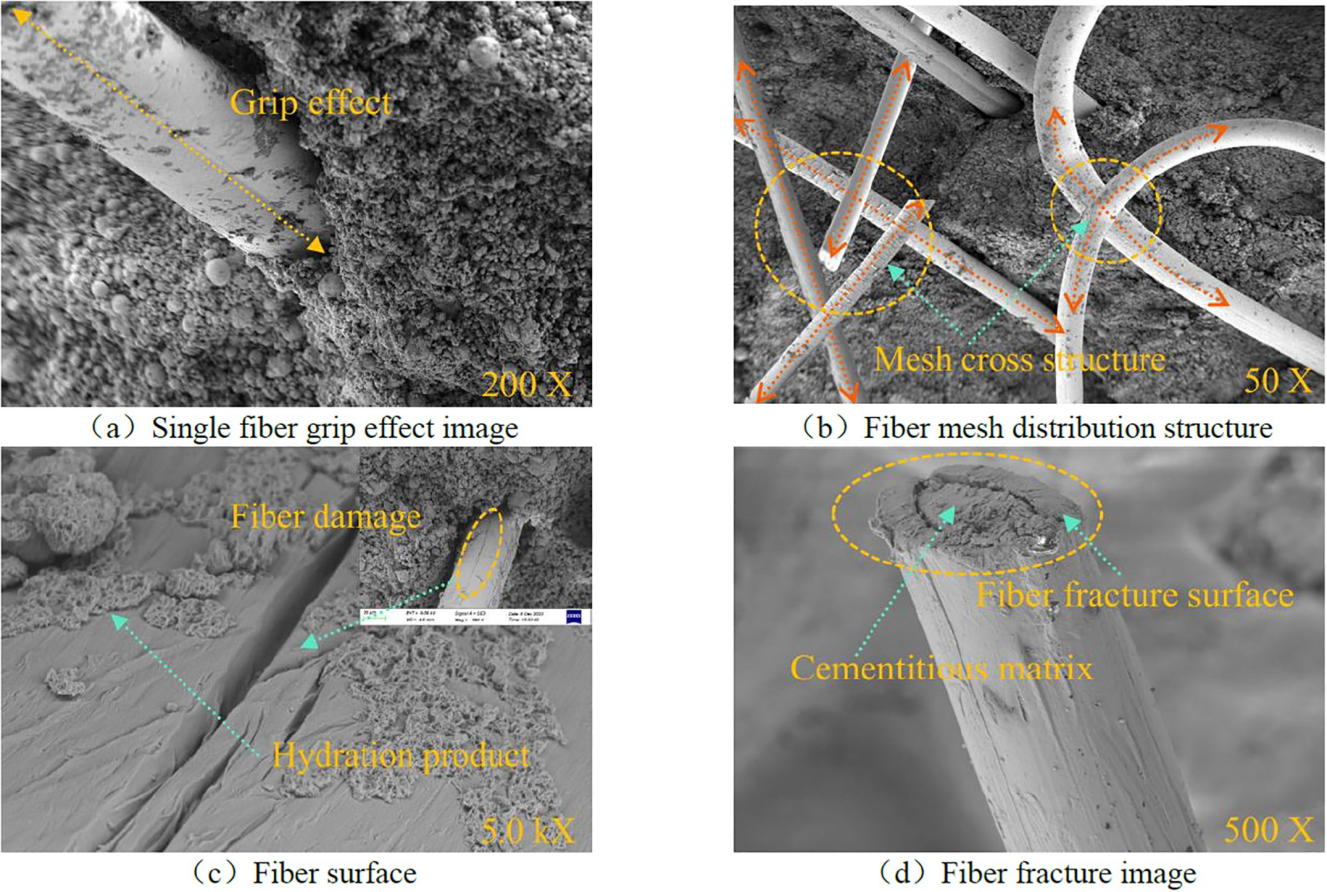
Fiber occurrence form and failure mode.

The fiber morphology and failure mode in the sample are shown in Fig 12. Fig 12(a) and 12(b) show that the fiber has two main modes of action inside the matrix. First is the single-fiber bonding effect. The fiber is independently distributed inside the sample, and it is bonded with the coal gangue particles by the hydration gel matrix. The contact interface between the two produces cohesion and forms an effective bonding effect. The hydration products stick to the fiber’s surface and unite with it inside the matrix, as seen in Fig 12(c). A significant number of hydration products build up to create a topology resembling a honeycomb mesh. Between the solid and hollow structures lies a continuous structure known as the honeycomb topology. The structure can spread and withstand external stresses coming from all directions while resisting them, maintaining consistent buffer strength and strong structural integrity. When the sample is subjected to an external load, the fiber delays the formation and development of microcracks inside the sample through the cohesion generated by the gel material and the friction generated by the interface interaction between the coal gangue particles. During the evolution process, the fiber surface is gradually damaged (Fig 12(c)), until the fiber reaches the ultimate tensile strength when the fracture occurs (Fig 12(d)) to achieve yield failure. The second involves the three-dimensional mesh traction effect. An appropriate amount of fibers is randomly distributed inside the sample, with obvious interlacing phenomena, forming a stable three-dimensional interwoven network structure. The hydration products formed during the curing process play a good bonding role in the heterogeneous solid waste matrix material, and stabilize the relative position of coal gangue particles and fibers. The interior of the sample is interwoven into a continuous and integral network structure with a dense interior. Therefore, it is difficult for the fibers and coal gangue particles to slide relative to each other when subjected to load, enhancing the integrity of the sample.

## 4. Analysis of damage evolution characteristics

### 4.1 Progressive destructive behavior

**Fig 13:**
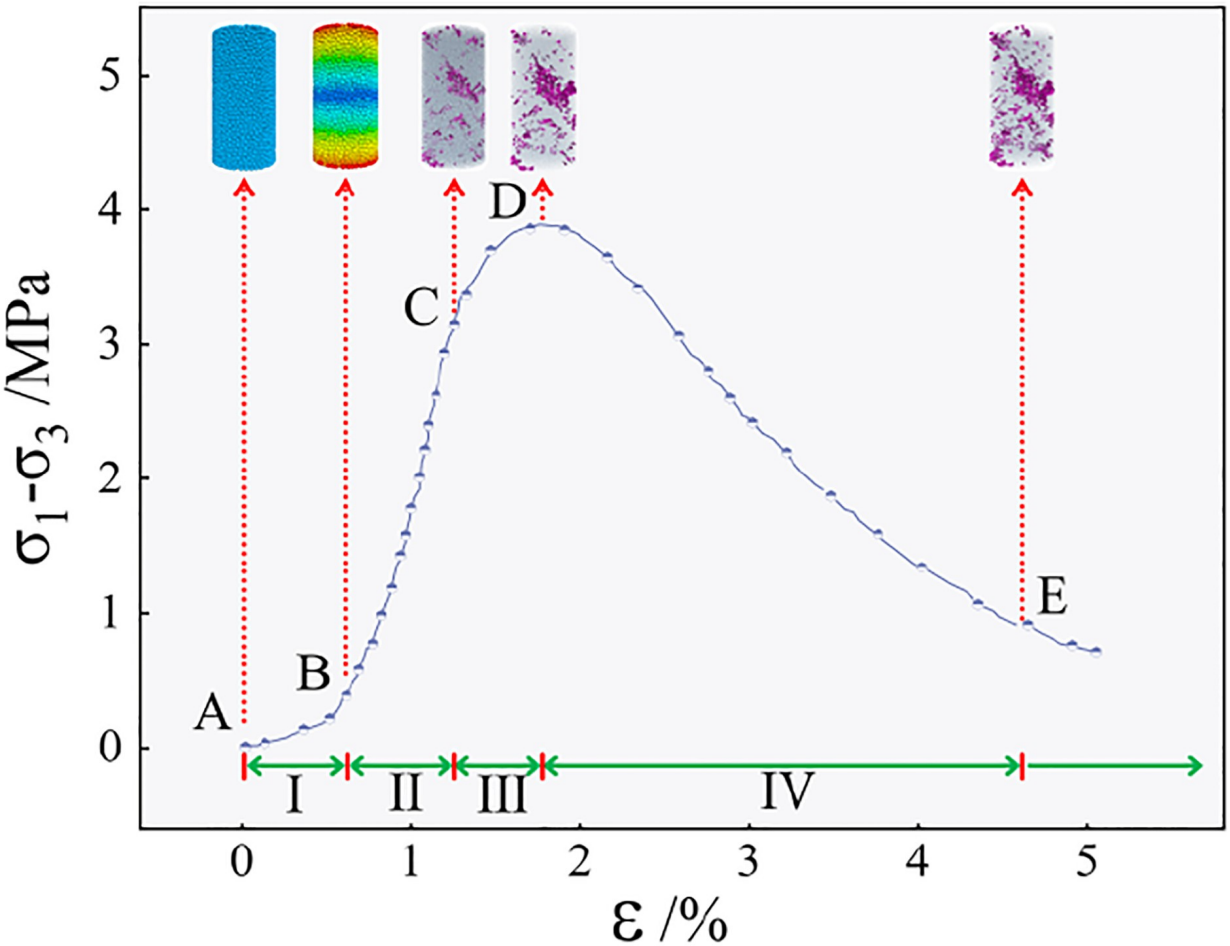
Typical deviatoric stress-strain curve of specimen.

In the triaxial compression process, failure of the specimen is not instantaneous, but the material damage behavior gradually evolves to the yield limit of the specimen. In this study, the deviatoric stress–strain curve is used to reflect the strength and deformation characteristics of the specimen under different loads. The typical deviatoric stress–strain curve of the paste filling material under triaxial compression is shown in Fig 13.

Under the condition of triaxial compression, the essence of the fracture of the fiber-reinforced ecological matrix cemented gangue gypsum filling material is the result of the initiation, expansion, and crack connection of the micro-cracks in the mixed matrix during the stress process, which is the macroscopic reflection of the deformation and failure accumulation of the micro-structure of the mixed matrix. According to the characteristics of the deviatoric stress–strain curve of the specimen, it is assumed that the entire failure process is divided into four stages, and the specific characteristics of each stage are as follows [38]. In the AB section, with the increase in the load applied to the specimen, the pore defects in the matrix are gradually compressed and compacted, and the volume of the specimen decreases with an increase in the load. The deviatoric stress–strain curve shows a nonlinear concave shape with a gradual increase in the tangent slope, which is the initial compaction stage. In the BC stage, the initial damage occurs in the sample, and the micro-voids in the matrix are connected and penetrated under the load and gradually evolve into micro-cracks. The deviatoric stress–strain curve is approximately linear, and the slope is defined as the elastic modulus of the sample. This stage is the stage of linear elastic damage development. In the CD section, deformation of the specimen continues forcefully, and the number of internal micro-cracks gradually increases. Based on continuous expansion and connection, the degree of damage and deterioration inside the matrix is clear. When the specimen reaches its ultimate strength, i.e., when the deviatoric stress reaches its maximum value, the deviatoric stress–strain curve exhibits a non-linear convex shape with a tangent slope; this stage is the plastic damage stage. In the DE section, the internal cracks of the sample increase sharply, crack development is fast, and clear shearing appears on the surface of the sample. The bearing capacity decreases continuously, tends to be stable, and finally dissipates to residual strength; this stage is characterized by instability and failure.

### 4.2 Establishment of damage evolution model

Owing to the particularity of the microstructure, the damage phenomenon in the fibre-reinforced ecological matrix cemented gangue gypsum filling material is extremely complex. The failure modes of composite materials are mainly divided into matrix cracking, interface weakening and separation, fibre fracture, etc. These failure modes are local anisotropic damage phenomena that often occur in samples. With the evolution of damage behaviour, the strength and stiffness of materials are reduced, and the bearing capacity of materials is affected. From Fig 13, it can be seen that the deformation of the paste filling material is nonlinear during the initial compaction stage of the sample. The development of matrix microcracks and the expansion of pore defects are the main reasons for the nonlinearity of the triaxial compression stress–strain curve of the fibre-reinforced ecological matrix cemented gangue paste filling material.

The traditional damage evolution model does not take into account the deformation characteristics of the eccentric stress-strain curve in the initial compaction stage, which makes it deviate greatly from the real compression curve. Therefore, the damage evolution model of the sample is divided into two parts in this paper.

According to the research results of references [39,40] and a large number of measured data, the deviatoric stress-strain relationship of the sample at the initial compaction stage can be expressed by Formula 5:

$$\sigma =\sigma_{A}{\left(\frac{\varepsilon }{\varepsilon_{A}}\right)}^{\text{4}}$$ (5)

In formula (5), *σ* represents the ultimate strength of the sample; *σ*_*B*_ is the deviatoric stress of the sample at the end of the initial compaction stage; *ε* is the strain of the specimen during triaxial compression; and *ε*_*B*_ is the strain of the filling body at the end of the initial compaction stage.

Due to the existence of micro-cracks, micro-cavities, and other defects, the mechanical properties of the sample are weakened under a certain load. In order to characterize this weakening effect, this paper defines a certain degree of weakening of paste filling materials as damage D. According to the equivalent strain hypothesis proposed by Lemaitre [41], the strain produced by the damaged material under the action of effective stress is equivalent to the strain produced by the material in the lossless state; that is, the constitutive model of the damaged material can be derived from the constitutive model of the lossless material, and the effective stress ${\tilde{\sigma }}_{ij}$ is used to represent the nominal stress *σ*. Therefore, the constitutive model of the sample can be expressed as:

$$\sigma ={\tilde{\sigma }}_{ij}\left(I-\left[D\right]\right)=\left[H\right]\left[\varepsilon \right]\left(I-\left[D\right]\right)$$ (6)

Where: *I* is identity matrix; [*D*] is damage variable matrix; [*H*] is the elastic modulus matrix of the sample; [*ε*] is the strain matrix.

The continuous medium model object is composed of material entities called material points. If the position occupied by the object in the three-dimensional Euclid space E^3^ is taken as the collection domain of material points, it is assumed that coal gangue particles, hydration products, and fiber material points are continuously distributed in the collection domain, so the fiber-reinforced ecological matrix cemented coal gangue paste filling material can be used as a continuous medium model. Under the general complex stress state, considering the influence of eccentric stress-strain peak value, synergistic effective area, and damage localization of paste filling materials, the damage constitutive relation of samples under triaxial compression can be expressed as follows:

$$\sigma ={\tilde{\sigma }}_{ij}\left(1-D\right)=E\;\varepsilon \left(1-D\right)$$ (7)

Where: *E* is the elastic modulus of the sample; *ε* is strain; *D* is the damage variable, when *D* = 0, the sample is in a state of no damage, and when *D* = 1, the sample is in a state of complete damage.

Assuming that the ultimate strength of the sample is related to the composition of the material, the distribution of internal defects, the grain size, and the types of hydration products, its strength can be expressed by statistical distribution. Assuming that the micro-cell strength of the sample obeys the Weibull distribution, the probability density function of the sample can be expressed by Eq 6:

$$P\left(F\right)=\frac{m}{F_{0}}\left(\frac{F}{F_{0}}\right)\text{exp}\left[-{\left(\frac{F}{F_{0}}\right)}^{m}\right]$$ (8)

Where: *P*(*F*) is the strength distribution function of the sample microcell; *F* is a random distribution variable of the strength of micro-cells; *m* and *F*_0_ are Weibull distribution parameters.

Assuming that the number of micro-elements whose cross section is damaged under a certain level of load is *N*_*Dn*_, the damage variable is defined as the ratio of the number of damaged micro-elements *N*_*Dn*_ to the total number of micro-elements *N*. The damage variable can be expressed by Eq 9:

$$D=\frac{N_{Dn}}{N}$$ (9)

Then, the area number of damaged microelements in any interval [*F*, *F* + d*F*] is *NP*(*Dn*)d*Dn*. When the load is loaded to a certain level *F*, the area number of damaged microelements can be expressed by Eq 10 as:

$$N_{Dn}\left(F\right)=\int_{0}^{F}NP\left(Dn\right)\text{dDn}=N\left\{1-\text{exp}\left[-{\left(\frac{F}{F_{0}}\right)}^{m}\right]\right\}$$ (10)

Substituting Eqs 9 into 10, the damage variable can be simplified as follows:

$$D=\frac{N_{Dn}}{N}=1-\text{exp}\left[-{\left(\frac{F}{F_{0}}\right)}^{m}\right]$$ (11)

Eq 11 is derived based on Weibull statistical theory and damage equation, and f value is the strength distribution variable of micro-element. Different damage models will be obtained when F takes different physical values. The failure of fiber-containing paste filling material is based on local tensile or shear failure and the result of crack propagation. Strain can better explain the whole process of crack propagation. Therefore, the strain is used as the distribution variable, and the damage evolution equation is:

$$D=1-\text{exp}\left[-{\left(\frac{\varepsilon }{F_{0}}\right)}^{m}\right]$$ (12)

The damage evolution model in the post-compaction stage can be obtained from Eqs 7 and 12, which can be expressed as:

$$\sigma =\sigma_{B}+E\left(\varepsilon -\varepsilon_{B}\right)\left\{\text{exp}\left[-{\left(\frac{\varepsilon -\varepsilon_{A}}{F_{0}}\right)}^{m}\right]\right\}$$ (13)

Suppose *σ*_*D*_ and *ε*_*D*_ are the ultimate stress and strain of the test. According to the geometric boundary conditions of the typical deviatoric stress-strain curve of the sample, at the peak point, the slope of the deviatoric stress-strain curve is zero, and it is obtained that:

$$\left\{\begin{array}{l} {\left.\sigma \right|}_{\varepsilon =0}=0\\ {\left.\sigma \right|}_{\varepsilon =\varepsilon_{D}}=\sigma_{D} \end{array}\right.,{\left.\frac{d\sigma }{d\varepsilon }\right|}_{\varepsilon =\varepsilon_{D}}=0$$ (14)

Substituting Formula 14 into Formula 13 gives:

$$\sigma_{D}=\sigma_{B}+E\left(\varepsilon_{D}-\varepsilon_{B}\right)\text{exp}\left[-{\left(\frac{\varepsilon_{D}-\varepsilon_{B}}{F_{0}}\right)}^{m}\right]$$ (15)

$$\frac{d\sigma }{d\varepsilon }\left|\left(\sigma =\sigma_{D},\varepsilon =\varepsilon_{D}\right)\right.=E\;\text{exp}\left[-{\left(\frac{\varepsilon_{D}-\varepsilon_{B}}{F_{0}}\right)}^{m}\right]\left[1-m{\left(\frac{\varepsilon_{D}-\varepsilon_{B}}{F_{0}}\right)}^{m}\right]=0$$ (16)

The expressions of Weibull distribution parameters *F*_0_ and *m* can be obtained by simplification:

$$F_{0}=\left(\varepsilon_{D}-\varepsilon_{B}\right)m^{\frac{1}{m}}$$ (17)

$$m=\frac{1}{\text{In}\left[\frac{E\left(\varepsilon_{D}-\varepsilon_{B}\right)}{\sigma_{D}-\sigma_{B}}\right]}$$ (18)

Therefore, the constitutive model of the post-compaction stage can be expressed as:

$$\sigma =\sigma_{B}+E\left(\varepsilon -\varepsilon_{B}\right)\text{exp}\left\{-\frac{1}{m}{\left[\frac{\varepsilon -\varepsilon_{B}}{\varepsilon_{D}-\varepsilon_{B}}\right]}^{m}\right\}$$ (19)

To sum up, the damage evolution model in the whole failure process of the test is obtained by combining Eqs 5 and 19:

$$\sigma =\left\{\begin{array}{ll} \sigma_{B}{\left(\frac{\varepsilon }{\varepsilon_{B}}\right)}^{\text{4}} & \left(\varepsilon <\varepsilon_{B}\right)\\ \sigma_{B}+E\left(\varepsilon -\varepsilon_{B}\right)\text{exp}\left\{-\frac{1}{m}{\left[\frac{\varepsilon -\varepsilon_{B}}{\varepsilon_{D}-\varepsilon_{B}}\right]}^{m}\right\} & \left(\varepsilon \geq \varepsilon_{B}\right) \end{array}\right.$$ (20)

### 4.3 Validation of damage evolution model

To validate Eq (20), the indoor triaxial compression test curve of the sample is compared with the theoretical curve. According to the deviatoric stress–strain curve obtained from the indoor triaxial compression test, the ultimate stress and ultimate strain values of the fibre-reinforced ecological matrix cemented gangue gypsum filling material are calculated, and the elastic modulus of each curve is calculated. Table 7 presents the parameters of the sample damage evolution model. (Owing to space limitations, only the fitting results of group A tests are presented).

**Table 7:** Sample damage evolution model parameters.

Sample number	Elastic modulus /MPa	*σ*_*B*_/MPa	*ε*_*B*_/%	*σ*_*D*_/MPa	*ε*_*D*_/%	m
A1	187.74	0.22	0.47	2.86	2.28	3.96
A2	208.53	0.41	0.87	3.32	2.52	5.97
A3	224.69	0.46	0.71	3.85	2.56	4.91
A4	242.08	0.69	0.97	4.67	3.41	2.53
A5	302.31	0.61	0.84	5.74	2.83	6.28
A6	399.65	0.72	0.98	7.48	2.93	7.03

**Fig 14:**
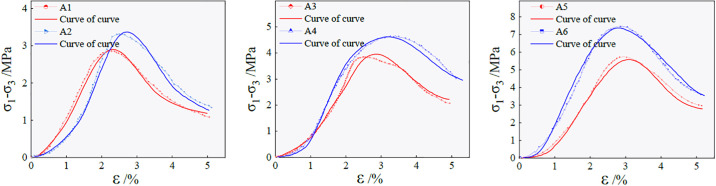
Triaxial compression test curves and theoretical curves of group A samples.

As shown in Fig 14, the theoretical and test curves are consistent in the elastic stage before the peak and differ slightly after the peak. The existing literature [42] modified the damage model of ordinary tailings cemented filling, but the results are not ideal. This indicates that the damage evolution model established by considering the nonlinear deformation characteristics of the initial compaction stage can better describe the deviatoric stress–strain relationship of the paste filling material during triaxial compression. Therefore, this model has reference value for engineering design and analysis.

## 5. Discussion

In this study, the triaxial compression performance and damage characteristics of fiber reinforced ecological matrix cemented gangue paste filling material were explored. Although the early research has discussed the research and development of geopolymer filling materials, most of them focused on the study of compressive properties, and did not explicitly discuss the shortcomings that fibers can improve the brittle failure and poor toughness of geopolymers. Moreover, the traditional damage constitutive model did not take into account the deformation characteristics of the initial compaction stage of the stress-strain curve of filling materials, which made it very different from the real compression curve.

Our research shows that adding 5‰ polypropylene fiber (12mm) into geopolymer cemented coal gangue material can significantly improve the shear performance of the sample, because a proper amount of fiber forms a three-dimensional network structure in the sample matrix, which effectively inhibits the process of matrix crack propagation and connection during loading. At the same time, we combined SEM-EDS and XRD to analyze the polymerization products. In alkaline environment, slag and fly ash produced a large number of hydrated calcium silicate and hydrated calcium aluminosilicate, which effectively bonded coal gangue particles. The piecewise damage evolution model can describe the stress-strain curve of the specimen during loading.

In this study, the triaxial compressive properties and damage characteristics of fiber reinforced ecological matrix cemented gangue paste filling materials are comprehensively discussed. However, we may need long-term research to grasp the essential reasons for the failure of fiber reinforced ecological matrix cemented gangue paste filling materials under complex loads in real environment. In the future research, we may explore the progressive failure mechanism of fiber-reinforced ecological matrix cemented gangue paste filling material in the actual working environment.

## 6. Conclusion

The mechanical properties of fiber-reinforced ecological matrix cement and gangue gypsum filling material were studied by a triaxial compression test, and the samples were characterized and analyzed using microscopic technology. The following conclusions were drawn:

1. Adding polypropylene fiber to the ecological matrix cemented gangue gypsum filling material can effectively improve the ultimate strength of the sample and the corresponding effective stress strength index. The ultimate strength and cohesion increase first and then decrease with the increase in fiber content. When the fiber content is 5 ‰, the two reach their maximum, the ultimate strength is 4.67 MPa, and the cohesion is 530.74 kPa. The elastic modulus fluctuates with the increase in fiber content, and the value is the smallest when the fiber content is 5 ‰. The ultimate strength and cohesion first increase and then decrease with the increase in fiber length, reaching their maximum when the fiber length is 12 mm. The elastic modulus gradually decreases and tends to be gentle with the increase in fiber length. There is no obvious relationship between the internal friction angle and the fiber parameters.
2. With the help of micro-scanning technology, it was found that the amorphous ecological glue condensation product formed by the reaction of slag and fly ash in the alkaline environment is filled between the coal gangue particles and fibers. The action mode of the fibers in the sample is mainly divided into single-grip anchoring and three-dimensional mesh traction. When crack initiation and development connections occur inside the matrix, the fibers produce a crack resistance effect. During the failure process, the brittle characteristics are improved by bridging, and the ductility of the ecological cementitious coal gangue matrix is enhanced.
3. Taking the end point of the initial compaction stage as the critical point, the deviatoric stress-strain curve is divided into the initial compaction stage and the damage evolution stage. According to the deformation characteristics of the sample before and after the critical point, a new damage evolution variable of the mixed material is used to establish a piecewise damage evolution model with the end point of the initial compaction stage as the critical point. The damage evolution model curves of the samples under different conditions are obtained. The theoretical model is verified by experiments. The experimental curves are in good agreement with the theoretical curves, indicating that the established theoretical model has certain reference value for the analysis and evaluation of the mechanical properties of paste filling materials.

The research results can help other scholars understand the stability and bearing capacity of fiber-reinforced ecological matrix cemented tailings paste filling materials, optimize the structure and performance of the filling body, reduce the use of materials, reduce the impact on the environment, and improve the efficiency of resource utilization. In the next stage, the author hopes to make more contributions to the durability of alkali-activated geopolymer filling materials.

## Supporting information

S1 File (ZIP)

S2 File (XLSX)

S3 File (XLSX)

S4 File (XLSX)

S5 File (XLSX)

S6 File (XLSX)
